# Assessment of Cerebrovascular Accident and Transient Ischemic Attack Risk Factors in Elderly vs. Non-Elderly Patients at a Tertiary Care Hospital in Eastern Province, Saudi Arabia

**DOI:** 10.7759/cureus.18391

**Published:** 2021-09-30

**Authors:** Ahmed S Mohammedin, Wesal S Horaib, Razan A Alshamsi, Sallumah O Alrashdi, Dalal A Aleidi, Mudhawi S Al Subaie, Noor-Ahmed Jatoi

**Affiliations:** 1 Geriatric Medicine, Ain Shams University Hospital, Cairo, EGY; 2 Geriatric Medicine, King Fahad Hospital of the University, Imam Abdulrahman bin Faisal University, Khobar, SAU; 3 Internal Medicine, King Fahad Hospital of the University, Imam Abdulrahman bin Faisal University, Khobar, SAU; 4 Vascular Medicine, King Fahad Hospital of the University, Imam Abdulrahman bin Faisal University, Khobar, SAU

**Keywords:** stroke complications, stroke risk factors, cerebrovascular accidents, stroke, transient ischemic attack, older adults

## Abstract

Background

The survivors of cerebrovascular accidents (CVA) or stroke are often left with several mental and physical disabilities which create a major social and economic burden. However, research addressing the risk factors of CVA and transient ischemic attacks (TIA), and their complications are insufficient.

Aim of the study

To assess the CVA and TIA risk factors (hypertension, diabetes mellitus type 2, dyslipidemia, coronary artery disease, atrial fibrillation, obesity, hypercoagulopathy, anti-platelet and anticoagulant use, carotid artery stenosis, and hypothyroidism) and complications (pneumonia, urinary tract infection and deep venous thrombosis) among a sample of elderly patients compared to non-elderly adult patients receiving care at King Fahd Hospital of the University in Al-Khobar, Saudi Arabia.

Methods

A retrospective observational study was conducted at King Fahd Hospital of the University in Al-Khobar, Saudi Arabia. Multiple risk factors and complications of CVA and TIA were retrieved from the medical records of the studied patients that fulfilled the inclusion criteria of patients diagnosed with CVA and TIA aged ≥ 60 years (elderly sample) and 18-59 years old (comparison non-elderly sample), who were followed up by internal medicine, neurology, and geriatric medicine departments. The total participant size was 259 patients, of which 149 were elderly.

Results

The occurrence of risk factors was more common in the senior age group. Hypertension was the most frequent risk factor in both age groups, while dyslipidemia, atrial fibrillation, and obesity were significantly associated with the development of CVA and TIA in the elderly. Moreover, post-CVA and TIA complications were more frequent in the group with elderly patients, with urinary tract infections being the most reported complication.

Conclusion

This study concluded that the most frequent risk factors were hypertension and type 2 diabetes mellitus. The findings of this study call for providing extra preventive care for elderly patients with dyslipidemia, atrial fibrillation, and obesity, and for more aggressive prevention of post-CVA and TIA complications in older age groups.

## Introduction

Cerebrovascular accident (CVA) or stroke can be defined according to World Health Organization (WHO) criteria as the sudden focal or global development of neurological deficit for more than 24 hours or leading to death without a clear cause other than a vascular origin [1]. It is classified into two main types: ischemic and hemorrhagic, where transient ischemic attack (TIA) is a transient episode of neurologic dysfunction caused by focal brain, spinal cord, or retinal ischemia, without acute infarction [2].

Stroke is considered one of the most common leading causes of death and disability globally [3]. According to the Global Burden of Disease Stroke Statistics Worldwide for the year 2016, there are 13.7 million new cases of stroke with a prevalence of 80.1 million cases. Furthermore, about 5.5 million deaths and 116.4 million disability‐adjusted life years (DALYs) were caused by strokes [4].

A systematic review conducted during the period of 2000 to 2014 revealed an incidence rate in the Middle East of 22.7 to 250/100,000 population per year [5]. Several studies conducted in Saudi Arabia showed an incidence rate of 30 to 40/100,000 population per year and prevalence of 186/100,000, with morbidity and mortality rates expected to be doubled by 2030 [6-7].

Furthermore, the risk of chronic diseases among the Saudi elderly population is high, with diabetes mellitus type 2 (DM-T2) and hypertension being among the five major medical conditions [8], which are risk factors for developing stroke.

In Saudi Arabia, a review of the recent literature found that hypertension was the major risk factor for stroke among the Saudi population [9]. Several global studies showed hypertension to be a leading risk factor for developing stroke and TIA [10-13]. Other similar results regarding hypertension were found in studies in the Middle East [14-15].

With regards to blood glucose levels, the prevalence of high blood glucose among stroke cases was diverse globally [11-12,14-15], while in Saudi Arabia, diabetes mellitus was reported to be the second most frequent risk factor [9].

Regarding body mass index, obesity was frequent in studies assessing stroke cases globally [10,12] which is consistent with the results of studies done in Saudi Arabia, where a study showed that 75% of ischemic stroke cases were obese [16-17].

Upon reviewing the literature there was limited information regarding the risk factors of developing CVA and TIA in the Eastern Province of Saudi Arabia, let alone studies focusing on investigating the risk factors in the elderly population. Therefore, research concerning the assessment of the CVA and TIA risk factors in elderly patients in the Eastern Province in Saudi Arabia is necessary to bridge the knowledge gap and improve the healthcare provided to this population and have clearer targets to direct the preventive measures.

The aim of this study was to assess the risk factors of CVA or stroke and TIA among a sample of elderly patients compared to non-elderly adult patients receiving care at King Fahd Hospital of the University in Khobar, Saudi Arabia. This is expected to help in establishing proper prevention programs.

## Materials and methods

This is a retrospective observational study conducted at King Fahd Hospital of the University (KFHU) in Al-Khobar, Eastern Province, Saudi Arabia. KFHU is a tertiary care and university hospital with 550 beds in addition to a day-care unit of 16 beds. The hospital has blended medical records, including both handwritten and electronic medical records. The database system uses the international classification of disease coding [18].

The total sample size was 259 patients (149 elderly and 110 non-elderly), and the elderly constituted 57.5% of the participants. The subjects were diagnosed with CVA and/or transient ischemic attack. The inclusion criteria were those aged 60 years and above (the elderly group) and 18-59 years old (the comparison non-elderly group). All patients were followed up by neurology, internal medicine, or geriatric medicine services during 2010-2020, including both Saudi and non-Saudi citizens. Patients with incomplete documentation were excluded. 

The demographic information (age, gender, nationality), TIA events, type of stroke, and risk factors such as age, dyslipidemia, hypertension, diabetes mellitus, obesity, smoking, cardiac diseases (including atrial fibrillation, coronary artery disease, valvular heart disease, cardiomyopathy), hypercoagulable state, antiplatelet and anticoagulant use, carotid stenosis, and hypothyroidism, were retrieved from the medical records of the study sample fulfilling the inclusion criteria for a duration of one month. Post-CVA and TIA complications (pneumonia, pressure ulcer, deep vein thrombosis, and urinary tract infections) were also retrieved. 

Institutional review board approval was obtained. All researchers followed the research ethics and hospital protocol. Data were extracted from the patients’ digital and scanned handwritten medical records. 

Data were then entered into Microsoft Excel, and statistical analysis was performed using statistical application software (SPSS version 26). The results of the categorical measurements are presented as percentages (%). P-value was assessed at a 5% significance level (P<0.05). The chi-square test was used to determine the significance of the categorical variables between the two groups.

There was some difficulty in obtaining all the required information from the digital medical record system due to incomplete documentation. This may be due to the use of a blended handwritten and digital system. For a more comprehensive evaluation of all risk factors, it was recommended that the missing information from the medical records be completed via contacting patients. Furthermore, although the results were significant, the small sample size questions the reliability of the significance. To confirm the significance of the results, a larger sample size is recommended. 

## Results

**Figure 1:**
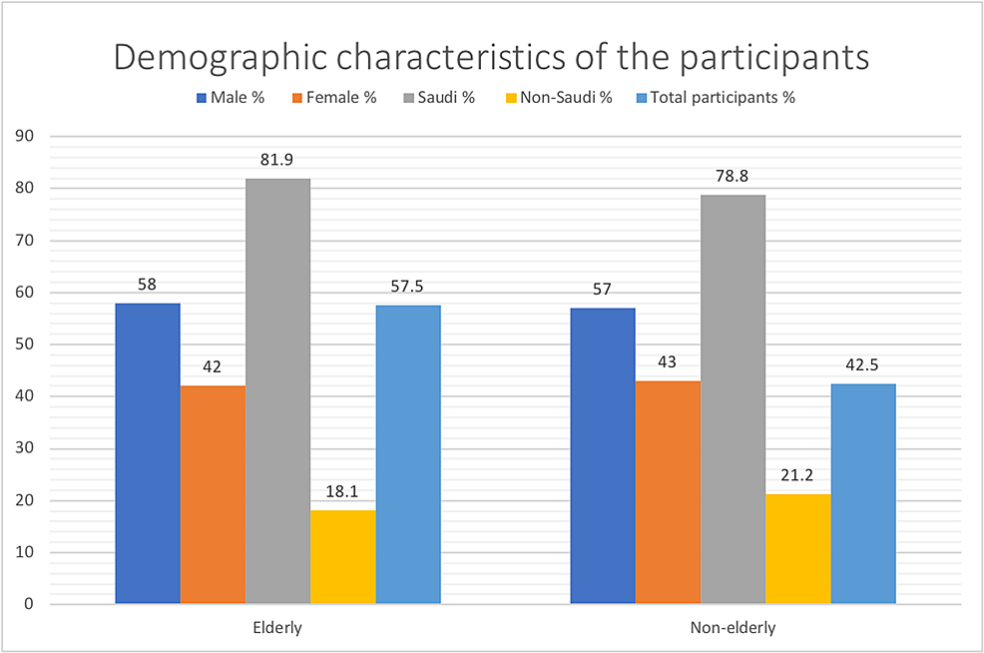
Demographic Characteristics of Participants Analyzed by Elderly and Non-Elderly patients (n = 259)

The elderly participants in this study were 57.5% while the non-elderly 42.5% of the sample size. The demographic characteristics of the participants including gender and nationality are described in Figure 1. Ischemic stroke and TIA were the most frequent conditions presented in patients who were 70-79 years of age (Table 1).

**Table 1:** Analysis of Cerebrovascular Accidents and Transient Ischemic Attacks by Age Group in Elderly Patients (n=149) N - Number.

Age groups (in years)	Cerebrovascular accidents and transient ischemic attack				
	Ischemic stroke	Transient ischemic attack (TIA)	Hemorrhagic stroke	Both (ischemic & hemorrhagic)	No stroke (only TIA)
60-69 N (%)	36 (24%)	23 (15%)	24 (16%)	4 (3%)	9 (6%)
70-79 N (%)	37 (25%)	28 (19%)	2 (1%)	3 (2%)	9 (6%)
80-89 N (%)	11 (7%)	10 (7%)	3 (2%)	2 (1%)	4 (3%)
more than 90 N (%)	2 (1%)	2 (1%)	1 (1%)	1 (1%)	1 (1%)

When compared between the non-elderly and elderly age groups, stroke and TIA were higher in the elderly patients. Moreover, the most affected age category in the non-elderly group was 50-59 years (49%), and in the elderly group 60-69 years (49%). TIA and hemorrhagic stroke were slightly more common in non-elderly than elderly (48.2% vs. 42.3% and 25.5% vs. 20.1%, respectively), while ischemic stroke was more frequent in the elderly (57.7% vs. 38.2%).

Risk factors 

The frequency of risk factors in elderly age groups is presented in Table 2. The most frequent risk factors were hypertension, DM-T2, dyslipidemia, coronary artery disease, and valvular heart disease, where the majority of the risk factors were commonly present in the 60-69-year and 70-79-year age groups. 

**Table 2:** Frequency of Risk Factors Analyzed by Age Groups in Elderly Patients (n=149) N - Number, PT - Prothrombin Time, aPTT - Activated Partial Thromboplastin Time, AF - Atrial Fibrillation, SVT - Supraventricular Tachycardia, CT - Computed Tomography.

Risk factors	Age groups (in years)			
	60-69	70-79	80-89	90 and above
Hypertension N (%)	59 (40%)	44 (30%)	18 (12%)	3 (2%)
Diabetes mellitus N (%)	52 (35%)	39 (26%)	10 (7%)	5 (3%)
Dyslipidemia N (%)	41 (27%)	38 (25%)	11 (7%)	1 (1%)
Coronary artery diseases N (%)	14 (9%)	18 (12%)	5 (3%)	0 (0%)
Valvular heart diseases N (%)	13 (9%)	6 (4%)	6 (4%)	0 (0%)
High level of PT^*^ and aPTT in hemorrhagic stroke N (%)	9 (6%)	2 (1%)	0 (0%)	1 (1%)
AF and SVT N (%)	9 (6%)	10 (7%)	8 (5%)	1 (1%)
Hypercoagulable state N (%)	9 (6%)	1 (1%)	1 (1%)	0 (0%)
Hypothyroidism N (%)	8 (5%)	6 (4%)	1 (1%)	1 (1%)
Antiplatelet use for hemorrhagic stroke N (%)	11 (7%)	4 (3%)	4 (3%)	0 (0%)
Anticoagulant use for hemorrhagic stroke N (%)	6 (4%)	3 (2%)	3 (2%)	0 (0%)
Congestive heart failure N (%)	5 (3%)	5 (3%)	4 (3%)	1 (1%)
Carotid stenosis by CT and duplex for internal and external branches N (%)	10 (7%)	8 (5%)	1 (1%)	1 (1%)
Heart block N (%)	2 (1%)	4 (3%)	1 (1%)	0 (0%)
Cardiomyopathy N (%)	3 (2%)	0 (0%)	0 (0%)	0 (0%)

With respect to the significant risk factors associated with stroke and TIA in the elderly age groups (Table 3), our analysis showed that dyslipidemia, atrial fibrillation, and obesity were statistically significant, (p<0.05). As for BMI, 42.3% of elderly patients were categorized as overweight and with class 1 obesity, while only 11.4% had normal weight. Although statistical analysis showed a significant difference, this needs further confirmation through a larger sample size.

**Table 3:** Association Between Risk Factors and Age Groups in the Elderly (n=149)

Risk factors		Age groups (in years)				Total (%)	Significance (P- value)
		60-69 (n=73)	70-79 (n=51)	80-89 (n=20)	90 and above (n=5)		
Gender							NS
	Male : Female (%)	64 : 36	49 : 51	60 : 40	60 : 40	58 : 42	
Dyslipidemia (%)		41 (56)	38 (75)	11 (55)	1 (20)	91 (61)	= 0.037
Hypertension		59 (80)	44 (86)	18 (90)	3(60)	124 (83)	NS
Diabetes (%)		52 (71)	39 (77)	10 (50)	5 (100)	106 (71)	NS
Atrial Fibrillation or Supra Ventricular Tachycardia (%)		9 (12)	10 (20)	8 (40)	1 (20)	28 (19)	= 0.048
Coronary Artery Disease (%)		14 (19)	18 (35)	5 (25)	-	37 (25)	NS
Hypothyroidism N (%)		8 (11)	6 (12)	1 (5)	1 (20)	16 (11)	NS
Body Mass Index (%)	Under	1 (1)	2 (4)	-	2 (40)	5 (3)	= 0.001
	Normal	5 (7)	7 (14)	5 (25)	-	17 (11)	
	Overweight	21 (29)	16 (31)	2 (10)	-	39 (26)	
	Class 1 obesity	14 (19)	4 (8)	6 (30)	-	24 (16)	
	Class 2 obesity	4 (6)	5 (10)	1 (5)	-	10 (7)	
	Class 3 obesity	4 (6)	4 (8)	-	-	8 (5)	
	Not available	24 (33)	13 (26)	6 (30)	3 (60%)	46 (31)	

When comparing between the elderly and non-elderly age groups (Table 4), hypertension was the most frequent in both age groups, followed by diabetes, dyslipidemia, and obesity. Around two-thirds of the elderly and 45.5% of the non-elderly with stroke and TIA had dyslipidemia, this difference was also statistically significant (p=0.015). Hypertension was more common in elderly patients who developed stroke and TIA (83.2%)than in non-elderly patients (70.6%), which was considered statistically significant (p=0.016). Furthermore, diabetes was more frequent in elderly patients who developed stroke and TIA (71.1%) compared to non-elderly patients, which was also statistically significant (p=0.003). As for the BMI, being overweight was the most common sub-category in both age groups. However, there was no statistically significant difference in BMI between the elderly and non-elderly with stroke and TIA.

**Table 4:** Association of Risk Factors and Post-CVA and TIA Complications With Age Groups in Elderly and Non-Elderly (159)

Risk factors			Age groups			Significance (P- value)
			Non-elderly (%)	Elderly (%)		
Gender		Male : Female	63 (57) : 47 (43)	87 (58) : 62 (42)		NS
		Dyslipidemia			50 (46)		91 (61)		= 0.015
Hypertension			77 (71)	124 (83)		= 0.016
Diabetes			58 (53)	106 (71)		= 0.003
Atrial Fibrillation or Supra Ventricular Tachycardia			4 (4)	28 (19)		= 0.000
Coronary Artery Disease			13 (12)	37 (25)		= 0.010
Hypothyroidism N (%)			7 (6)		16 (11)	NS
Body Mass Index	Under		1 (1)		5 (3)	NS
	Normal		13 (12)		17 (11)	
	Overweight		27 (25)		39 (26)	
	Class 1 Obesity		21 (19)		24 (16)	
	Class 2 Obesity		11 (10)		10 (7)	
	Class 3 Obesity		7 (6%)		8 (5)	
	Not available		29 (27)		46 (31%)	
Number of Complications	0		95 (87)		99 (66)	0.000
	1		9 (8)		44 (29)	
	2		3 (3)		6 (4)	
	3		2 (2)		-	
Pneumonia			7 (6)		9 (6)	NS
Pressure Ulcer			1 (1)		2 (1)	NS
Deep Venous Thrombosis			1 (1)		1 (1)	NS
Urinary Tract Infection			12 (11)		44 (30)	0.000

About 18.8 % of the elderly who developed stroke and TIA had atrial fibrillation, while this was only seen in 3.7% of the non-elderly group (p=0.000). Coronary artery disease was more common in the elderly group (24.8%) than in the non-elderly (11.9%)(p=0.000); all were statistically highly significant. 

Moreover, the frequency of hypercoagulopathy was similar between the two groups. Internal carotid stenosis was approximately 6% in the elderly and 5.5% in the non-elderly. However, external carotid stenosis was five times more common in the elderly (4.7% vs. 0.9%). All cardiac diseases, including atrial fibrillation, supraventricular tachycardia, coronary artery disease, valvular heart disease, congestive heart failure, and heart block were more common in the elderly, except for cardiomyopathy, which was common in non-elderly patients (5.5% vs. 2%). Approximately 11% of the elderly and 6.4% of the non-elderly with stroke and TIA had hypothyroidism; the difference was not considered statistically significant* *(Table 4).

Complications 

Comparing frequencies between the age groups shows that most patients had no complications (66.4% in the elderly and 87.3% in the non-elderly)(Table 4). This was followed by the presence of one complication (29.5% vs. 8.2%) in elderly and non-elderly patients, respectively. However, the low number of complications could be explained by possible under-reporting. All complications were more common in the elderly group, except for pneumonia and deep vein thrombosis (DVT), which were almost the same between both age groups. Overall, the most common complication was urinary tract infection (UTI), which was more frequent in the elderly (29.5% vs. 10.9%), and the least common complication was DVT (0.7% vs. 0.9%).

Moreover, more post-CVA and TIA complications were related to the elderly group compared to the non-elderly group. In general, one-third of the elderly group (33.5%) developed one to two post-stroke complications. Although only 12.9% of non-elderly patients developed one to three complications following a stroke, it was statistically significant (p=0.0001). With respect to each complication, all were more common among the elderly, but there was no statistically significant difference with the non-elderly group; however, around one-third of the elderly patients developed UTI as a post-stroke complication, while only 11% of the non-elderly patients developed UTI. This difference was statistically significant(p=0.0001). 

In regards to post-CVA and TIA complications in relation to age among the elderly, Table 5 shows that the percentage of patients who had more than one complication after CVA and TIA was 43.1% in the group aged 70-79 years, compared to 17.8% in the group aged 60-69 years (p=0.049). Moreover, all the complications were more frequent among the elderly age group. 

**Table 5:** Post-CVA and TIA Complications Categorized by Age in Elderly Patients (n=149)

Variables		Age groups (in years)				Total (n=149)	Significance (P-value)
		60-69 (n=73)	70-79 (n=51)]	80-89 (n=20)	90 and above (n=5)		
Number of complications (%)	0	55 (75)	28 (55)	12 (60)	4 (80)	99 (67)	= 0.049
	1	13 (18)	22 (43)	8 (40)	1 (20)	44 (30)	
	2	5 (7)	1 (2)	-	-	6 (4)	
Pneumonia (%)		5 (7)	3 (6)	-	1 (20)	9 (6)	NS
Pressure ulcer (%)		2 (2)	-	-	-	2 (1)	NS
Deep vein thrombosis (%)		1 (1)	-	-	-	1 (0.7)	NS
Urinary tract infection (%)		15 (21)	21 (41)	8 (40)	-	44 (30)	= 0.026

## Discussion

In our study, ischemic stroke was generally more common than hemorrhagic stroke, even in the young age group. The same finding was reported in other studies from Sudan [19] and Nigeria [20].

Our results have shown the highest frequency of stroke among the elderly age group (especially the 60-69 age sub-group), which is consistent with the results of other studies conducted in Saudi Arabia to assess first stroke events; the mean age of the first stroke among Saudis was 63 years, while this tends to be 69 years in the United States of America (USA) and 70 years in the United Kingdom (UK) [6]. 

As for the BMI in this study, most of the patients were found to be overweight in both age groups. A study was conducted to assess the relationship between ischemic stroke and abdominal obesity concluded that people with a waist-to-hip ratio (WHR) equal to or higher than the median had an increased risk of stroke even when adjusting for other risk factors [21]. 

The frequency of DM varied between the results of our study and the Sudanese one (71.1% in the elderly and 53.6% in the non-elderly vs. 8% in the older age group and 11.2% in the younger age group, in our study and the Sudanese one, respectively). Obesity was the fourth most common risk factor in our study compared to the fifth most common risk factor in Sudanese study, which is close in rank. However, the frequency of obesity in Sudan (2.1% and 3.4%) was much lower than the frequency in our study (28% and 35%) in older and younger patients, respectively, which could also be explained by the higher economic status in Saudi Arabia [19]. 

Our study reported that dyslipidemia, AF, and obesity are statically significant in relation to CVA in the elderly age group, which is consistent with previous studies [16-17]. However, diabetes, hypertension, coronary artery disease, and hypothyroidism were found to be insignificant risk factors that were not concomitant with other findings. This may be due to the small sample size. Dyslipidemia was reported to be the top risk factor for CVA in a study conducted in China [22], and was also reported to be a significant risk factor in the elderly Saudi population [17]. According to a Chinese study, dyslipidemia was found to be a significant risk factor for CVA in elderly women than in men [22]. 

AF was more frequent in the age groups above 45 years in both stroke types [19]. Moreover, AF was a major significant risk factor in Saudi Arabia [8]. There was no significant difference in AF according to sex in patients aged 75 years and above [22]. A study in Chile showed that the frequency of AF in patients with ischemic stroke was similar to previous findings [23]; however, AF was more associated with females in another study conducted in China [12]. AF is also the most frequent risk factor for ischemic stroke among people with multiple vascular risk factors [10]. 

This study shows that factors such as dyslipidemia, hypertension, diabetes mellitus, AF, SVT, and coronary artery disease (CAD) presented frequently in elderly patients who developed CVA in the last 10 years (2010-2020) than in the non-elderly group. This means that these risk factors are more frequently associated with CVA in elderly patients than in non-elderly patients. Moreover, hypertension was the most frequent risk factor in both age groups. However, the Sudanese study showed that most of the risk factors, specifically hypertension and diabetes mellitus, were more frequent among those aged 15-45 years with CVA [19]. This may be due to the variation in socioeconomic status, lifestyle, preventive measures, and the quality of health care services in both populations that impact chronic disease control and sequelae.

In our study, urinary tract infection (UTI) was the most common complication, followed by pneumonia in both age groups. Pressure ulcers and deep vein thrombosis (DVT) were only reported in patients aged 60-69 years (1.3% and 0.7%, respectively). Similarly, one study that was conducted to assess complications after CVA showed low frequencies of DVT and pulmonary embolism, and a high frequency of UTI and pneumonia [24].

Several studies have shown that systemic hypertension, dyslipidemia, diabetes mellitus, heart disease, and atrial fibrillation (AF) are the most common modifiable risk factors for stroke and TIA, and these risks might be attributed partially to the lifestyle of the Saudi population. Changes in lifestyle are responsible for the higher frequency of modifiable risk factors of stroke, such as dyslipidemia and obesity. Therefore, urgent intervention and effective awareness regarding modifiable risk factors of CVA is needed [9]. 

## Conclusions

This study focused on the assessment of CVA and TIA risk factors and complications among elderly and non-elderly patients, the results concluded that the most frequent risk factors were hypertension and diabetes mellitus type 2, while dyslipidemia, atrial fibrillation, and obesity were significantly associated with the development of CVA and TIA in the elderly and overall, the risk factors and post-CVA and TIA complication were more frequent in the elderly group. 

These findings call for providing extra preventive care for patients with dyslipidemia, AF, and obesity, and for more aggressive prevention of post-CVA and TIA complications in older age groups. We would like to highlight the importance of accurate documentation in medical records of the missing data observed in our study: smoking, BMI, and stroke territory.
